# Phenolic, Carotenoid and Saccharide Compositions of Vietnamese *Camellia sinensis* Teas and Herbal Teas

**DOI:** 10.3390/molecules26216496

**Published:** 2021-10-27

**Authors:** Danh C. Vu, Sophie Alvarez

**Affiliations:** 1 Institute of Applied Technology, Thu Dau Mot University, Thu Dau Mot City 820000, Binh Duong, Vietnam; 2 Proteomics and Metabolomics Core Facility, Center for Biotechnology, University of Nebraska, Lincoln, NE 68503, USA; salvarez@unl.edu

**Keywords:** phenolic, carotenoid, jasmine tea, green tea, Vietnamese tea, artichoke

## Abstract

Tea (*Camellia sinensis*) and herbal tea have been recognized as rich sources of bioactive constituents with the ability to exert antioxidant actions. The aims of this study were to analyze phenolic, carotenoid and saccharide contents in a set of Vietnamese tea and herbal tea and compare the results with those of green and black teas marketed in the U.S. In total, 27 phenolics, six carotenoids and chlorophylls, and three saccharides were quantitatively identified. Catechins, quercetin glycosides and chlorogenic acid were the predominating phenolics in the teas, with the concentrations following the order: jasmine/green teas > oolong tea > black tea. Lutein was the dominant carotenoid in the teas and its concentrations were generally found to be higher in the jasmine and green teas than in the oolong and black teas. The study showed that the green teas originating in Vietnam had much higher levels of phenolics and carotenoids than their counterparts stemming from another country. The application of partial least squares discriminant analysis (PLS-DA) as a chemometric tool was able to differentiate phenolic profiles between methanolic extracts and tea infusions. Through principal component analysis (PCA), the similarities and dissimilarities among the jasmine, green, oolong, black teas and herbal teas were depicted.

## 1. Introduction

Tea, prepared from leaves and buds of *Camellia sinensis*, is among the most consumed beverages in the world. Herbal teas are beverages that are brewed from aerial parts or roots of plants, other than *Camellia sinensis,* with medicinal properties. Research has provided evidence that drinking tea and herbal tea is linked with multiple health benefits, including prevention of cardiovascular diseases, delay of the onset of neurodegenerative disorders, and protection against the development of certain cancers [1]. These health-promoting properties are partly attributed to the presence of a diverse mixture of phytochemicals, notably phenolics, in tea. Phenolics are a class of compounds with molecules comprising one or more aromatic rings bonded to hydroxyl groups. They are divided into different subclasses, such as phenolic acid, flavonoid, stilbene, coumarin and tannin. Evidence has shown that flavonoids are able to act as antioxidants, countering damaging effects of reactive oxygen species (ROS) [2]. The inhibition of multiple ROS-induced process steps in metabolic pathways in living cells results in reduced risks of certain cancers, such as prostate and breast cancers [3,4,5]. The presence of ROS initiates oxidative stress which plays a role in promoting events related to neurodegenerative diseases [6]. Antioxidants, such as flavonoids, may aid in improving endogenous oxidative stress-attenuating systems, contributing to prevention of deteriorations in cognitive performance. In addition to phenolics, alkaloids, such as caffeine and theobromine, amino acids, fiber, protein, saccharides, pigments and minerals in tea have been documented [7]. Carotenoids constitute a class of lipophilic pigments found in plants. They are known to play an important role in the formation of tea aroma during processing, and some of them are deciding factors for the quality of tea [8]. Evidence suggests the consumption of diets rich in carotenoids may be associated with reduced risks for cardiovascular diseases, diabetes and certain cancers [9]. Saccharides greatly affect the taste and smell of tea products [10]. Besides, saccharides, such as glucose, fructose, arabinose and xylose, may exert powerful actions on human immunity due to their benefits on the growth of bifidobacteria [11].

The tea cultivation in Vietnam springs from a long tradition just as in China and other East Asian countries. Some local legends recount that tea was first brought to Vietnam more than 1000 years ago [12]. Currently, Vietnam is one of the largest tea-producing countries in the world. During the period from 2017 to 2018, it exported 158,340 tons of tea, earning 265.9 million USD in revenue [13]. Vietnamese tea can be categorized into four popular varieties: green tea, oolong tea, black tea and scented tea. Green tea is made from tea leaves and buds that are heated after picking to preclude the enzymatic oxidation of polyphenols. During black tea production, tea leaves have undergone full oxidation and thus have a darker appearance and a stronger flavor. Oolong tea is a product which is produced with partial oxidation. Adding flowers, herbs or fruits to tea is used in making scented tea. Prior research reported that Vietnamese tea contained catechin and catechin derivatives at comparable levels with those obtained from China, Japan and Australia [14]. Recent case-control studies have indicated frequent consumption of Vietnamese tea may have positive impacts upon Vietnamese adults with type 2 diabetes [15] and men with prostate cancer [16]. Beyond these, data about bioactive phenolic contents and the potential health-promoting properties of Vietnamese tea are very limited, while those of the Chinese and Japanese have been well recorded.

The aim of this study was to quantitatively identify phenolic, carotenoid and saccharide composition in different teas manufactured in Vietnam. In addition, two herbal teas, including an artichoke tea and a refreshing tea prepared from *Senna obtusifolia*, *Glycyrrhiza uralensis* and *Sophora japonica*, were included in the study. Attempts to analyze these constituents in teas have been made to gain a better understanding of phytochemical composition and how these constituents contribute to the health benefits of tea consumption.

## 2. Materials and Methods

### 2.1. Chemicals and Reagents

Analytical phenolic standards (caffeic acid, cinnamic acid, chlorogenic acid, daidzein, (−)-epicatechin, ferulic acid, gallic acid, genistein, hesperetin, *p*-coumaric acid, *p*-hydroxybenzoic acid, kaempferol, phloretin, procyanidin A2, procyanidin B2, protocatechuic acid, quercetin, quercetin-3-O-glucoside, quercetin 3-O-galactoside, resveratrol, rutin, syringic acid and vanillic acid) were obtained from Sigma-Aldrich (St. Louis, MO, USA). The (+)-catechin hydrate, cyanidin chloride and delphinidin chloride were purchased from Cayman Chemicals (Ann Arbor, MI, USA). Acacetin, apigenin, luteolin, LCMS-grade methanol, acetone (ACS grade), naringenin and naringenin chalcone were purchased from Fisher Scientific (Pittsburgh, PA, USA). Ultra-pure water (with a resistivity of 18 MΩ.cm and a TOC level < 5 ppb) for all the experiments was prepared from a Milli-Q purification system (Millipore, MA, USA).

### 2.2. Tea Samples

**Figure 1:**
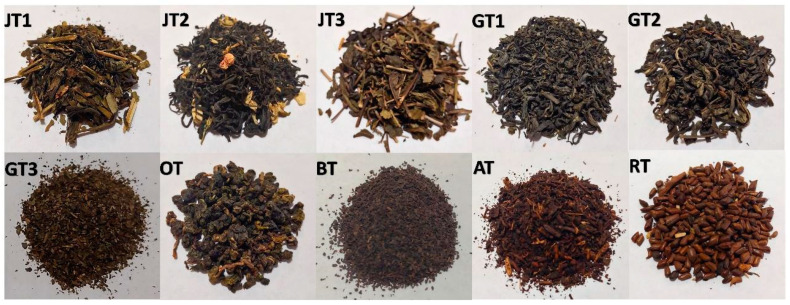
The tea and herbal tea samples used in the study.

Seven Vietnamese and three U.S. products were used in the study (Table 1, Figure 1). The Vietnamese products were purchased from tea manufacturers located in Vietnam, including two jasmine teas, two green teas, one oolong tea, one refreshing tea and one artichoke tea. The U.S. teas (one decaffeinated black tea, one decaffeinated green tea and one jasmine tea) were obtained from local groceries in Lincoln, Nebraska, USA. All the samples were stored in a freezer (−20 °C) until analysis.

### 2.3. Analysis of Phenolics

Each ground tea sample (approximately 100 mg) was weighed into a 2 mL Eppendorf tube, then extracted with 1.5 mL of 100% methanol. Two stainless steel beads (4.8 mm diameter, Biospect Products, OK, USA) were added. The mixture was homogenized in a TissueLyzer II (Qiagen, Hilden, Germany) at 20 Hz for 10 min and then centrifuged at 21,120× *g* for 5 min. The supernatant was collected and transferred to an HPLC vial. The tea samples were also extracted with 1.5 mL of boiling water (Milli-Q water) using the same extraction method. Both the methanolic and aqueous extracts were analyzed using liquid chromatography-tandem mass spectrometry (LC-MS/MS).

Analysis of phenolics in the tea samples was conducted using a Shimadzu Nexera X2 high performance liquid chromatography system coupled with a Sciex QTRAP 6500+ mass spectrometer (Sciex, MA, USA). The chromatographic separation of the phenolics was performed by an Agilent Eclipse XDB C18 (100 mm × 3.0 mm; 3.5 μm particle size) reverse-phase column (Agilent Technologies, CA, USA). The mobile phases consisted of 2% acetic acid in water (A) and 100% acetonitrile (B). The gradient conditions were 0–0.1 min, 6% B; 0.1–5 min, 6–17% B; 5–8 min, 17–20% B; 8–16 min, 90% B; 16–18 min, 90% B; 18–19 min, 6% at a flow rate of 0.4 mL/min. The injection volume was 1 µL. The column compartment was set at ambient temperature. The MS/MS system was operated with the IonDrive^TM^ Turbo V ion source (ESI) in positive and negative ion modes (Sciex, MA, USA). The ESI source operation parameters were as follows: source temperature, 450 °C; ion spray voltage, 5500 and –4500 V; ion source gas 1, ion source gas 2 and curtain gas, 50, 50 and 25 psi, respectively; collision gas (CAD), medium. The MS/MS system was operated in the multiple reaction monitoring (MRM) mode with the optimized collision. The ionization energy, MRM transition ions (molecular and product ions), collision energy (CE), declustering potential (DP), entrance potential (EP) and collision cell exit potential (CXP) were optimized by a Sciex Analyst software package (Table S1, Supplementary material). Analytical data were processed using Analyst 1.6.3 software platform (Sciex, MA, USA).

### 2.4. Analysis of Carotenoids

Carotenoids in tea were extracted following the method previously described by Dautermann and Lohr (2017) [17]. Approximately 150 mg of each ground tea sample were weighed into a 2 mL Eppendorf tube. Two stainless steel beads (4.8 mm in diameter) and 250 µL of acetone were added. The mixture was homogenized for 10 min at 20 Hz using the TissueLyzer II, then centrifuged for 5 min at 16,000× *g*. The supernatant was transferred into an LC vial prior injection into an ultra-performance liquid chromatograph (UPLC). All the sample preparation steps were performed under yellow light to protect carotenoids from UV degradation.

Carotenoids in tea samples were analyzed using an Agilent 1290 Infinity II UPLC system coupled with a 1290 Infinity II diode array detector (DAD). The system was equipped with an Acclaim C30 reverse-phase column (150 mm × 2.1 mm; 3 μm particle size). The mobile phases consisted of (A) acetonitrile/methanol (2:1, *v/v*) and (B) methanol/ethyl acetate (1:1, *v/v*). The gradient conditions were as follows: 0–2 min, 100%A; 8 min, 95%A and 5%B; 10–14 min, 85%A and 15%B; 14.5 min, 95%A and 5%B; 20 min, 100%A. The flow rate was 0.3 mL/min and column temperature was set at 30 °C. Detection was set at 440 and 450 nm. The injection volumes for carotenoid standards and tea extracts were 2 and 1 µL, respectively. The processing of chromatographic data was performed using OpenLab ChemStation software platform (Agilent Technologies, CA, USA).

### 2.5. Analysis of Saccharides

Extraction of saccharides in ground tea samples was conducted in the same way as that of phenolics. The supernatant was evaporated until dry using a SpeedVac vacuum concentrator (ThermoFisher Scientific, CA, USA). The pellet was resuspended in 200 µL of Milli-Q water. The mixture was shaken for 5 min and centrifuged at 21,120× *g* for 5 min. The soluble saccharides were analyzed using a 1260 Infinity ELSD (evaporative light scattering detector) coupled with an Agilent 1290 Infinity II UPLC system equipped with a Shodex Sugar SP0810 (300 mm × 8.0 mm ID) column. A supply of oil-free clean nitrogen was used to operate the detector. The mobile phase was 10% acetonitrile. Flow rate was set at 0.4 mL/min and column temperature was 80 °C. Total runtime was 35 min. The data processing was performed using OpenLab ChemStation software platform.

### 2.6. Statistical Analyses

All the experiments were conducted in triplicate. The results were shown as mean ± standard error. The quantitative data generated were analyzed using one-way analysis of variance (ANOVA) to determine variations in chemical composition between the tea products. Comparisons of means were drawn using XLSTAT software (XLSTAT Premium 19.5, Addinsoft, Paris, France). The Tukey’s Studentized Range HSD test was used to determine multiple comparisons at a significant level of *p* < 0.05.

Principal component analysis (PCA) was used to examine patterns in composition data and to highlight similarities and dissimilarities in phytochemical contents of the tea products. Partial least squares discriminant analysis (PLS-DA) was a suitable tool for diagnosing differences in tea phenolic compositions obtained between two extraction methods (methanol vs. water). Heatmap was used to graphically present tea chemical data. The PCA, PLS-DA and heatmap were implemented using MetaboAnalyst 4.0, a web-based metabolomics and statistical software platform [18].

## 3. Results and Discussion

### 3.1. Phenolic Contents in the Teas and Herbal Teas

In total, 30 phenolics, including phenolic acids, flavonoids, chalconoids and stilbenoids, were screened. The results of molecular ions, product ions and optimized ionization parameters of the screened phenolics are summarized in Table S1 (Supplementary material). All these compounds produced symmetric chromatographic peaks with widths less than 0.2 min. Gallic acid was the first phenolic compound to be detected (at 1.69 min) while acacetin eluted at as late as 13.60 min (Table S2, Supplementary material). The linear regression was successfully used to fit every calibration dataset over the concentration range, and all the constructed calibration equations had correlation coefficients (R^2^) greater than 0.9980. Estimation of limit of detection (LOD) and limit of quantification (LOQ) was based on signal-to-noise ratios of three and ten, respectively. The LOD and LOQ values ranged from 0.01 to 0.75 and from 0.03 to 2.47 µg/L, respectively. The data about calibration, sensitivity and repeatability are shown in Table S2 (Supplementary material). The results showed that the LC-MS/MS method used in this study offered sensitivity and selectivity needed for rapid and accurate quantification of phenolics in organic-rich matrices.

The results from the methanol extraction yielded evidence that 27 phenolics were present in the studied samples. The identification of these compounds was performed by comparing their retention times, MS and MS^2^ mass spectral data with those of the authentic analytical standards. The phenolic acid and flavonoid contents in the tea and herbal tea samples are shown in Table 2 and Table 3. Among the compounds detected, fifteen were found to be present in all the tea and herbal tea samples. Quercetin glycosides (i.e., quercetin glucoside, quercetin galactoside and rutin), catechin, epicatechin, chlorogenic acid and gallic acid predominated over the others. The green teas and jasmine teas were the groups of products that contained high levels of these compounds.

### 3.2. Flavonoids

Reportedly, catechin, epicatechin and procyanidins were commonly found as major tea constituents [19]. The mean concentrations of catechin (364.68–636.58 µg/g) and epicatechin (319.09–546.77 µg/g) in the jasmine teas were higher compared to the other teas. Along with the jasmine teas, the green tea samples were found to contain significantly higher levels of catechins than the studied black tea and the herbal teas (i.e., AT and RT). As shown in Table 2, the concentrations of catechins in the green teas are approximately 4–15 times as high as those in the black tea, respectively. This could be due to extensive enzymatic oxidation during production explaining the low amount of catechins in the black tea. The enzymatic process could also be responsible for the lower levels of catechins in the examined oolong tea compared to the jasmine and green teas. Indeed, in a review commenting on the health benefits of tea, Muhammad and Dickinson (2019) stated that catechins were relatively unstable and susceptible to oxidation, explaining a higher abundance of these constituents in green tea compared to oolong and black teas [20]. The amounts of catechins in the jasmine, green and oolong teas determined in the present study are comparable to those reported in prior research [21,22]. These studies have similarly shown that the order for catechin levels in tea samples is as follows: jasmine/green teas > oolong tea > black tea. This has also been demonstrated by a recent investigation into Chinese tea infusions by Pinto et al. (2020) [23]. Procyanidin B2, which is one of the important dimeric catechins in tea, was detected in all the samples, except for the artichoke tea, while procyanidin A2 was found in only GT3. The levels of procyanidin B2 were highest in the jasmine/green teas and lowest in the black tea (Table 2). Prior research revealed that this compound had much lower contents in black tea compared to those in green tea [24] and this was likely due to its breakdown during the withering and fermentation processes. A comparison among the green teas indicates that GT1 and GT2 derived from Vietnam-grown tea leaves contained higher amounts of catechins and catechin derivatives than GT3 produced from China-grown tea leaves.

Along with catechins, quercetin glycosides are mostly reported to be present in tea. The levels of quercetin glucoside and rutin were found higher in the jasmine and green teas than in the black tea while that of quercetin galactoside was the greatest in the oolong tea. Similar to catechins discussed above, quercetin glycosides had higher concentrations in GT1 and GT2 than GT3.

It is noted that there has been disagreement among the previous reports on concentrations of quercetin glycosides in tea. For example, Wu et al. (2012) and Jeszka-Skowron et al. (2018) revealed higher abundances of rutin and quercetin galactoside in Chinese green tea while Jiang et al. (2015) documented greater levels of these glycosides in Chinese black tea [21,25,26]. These remarkable variations could be due to harvesting, postharvest handling practices and tea processing [27,28]. In addition to catechins and quercetin glycosides, ten other flavonoids were detected and quantified in the present study (Table 2). These compounds were found as minor phenolics in the studied teas. Of these, apigenin, kaempferol, luteolin, naringenin and quercetin were present in all the samples. Previously, these flavonoids were also reported to be detected in tea at very low levels [29,30,31].

Among the studied teas, AT and RT demonstrated substantial differences in concentrations of catechins and procyanidins in comparison with the other teas. The results indicated considerably low levels of these compounds in RT (0.2–1.12 µg/g). Some compounds, such as catechin and procyanidin B2, were even undetected in AT. By contrast, the level of quercetin glucoside in RT (113.84 µg/g) was comparable to that of the black tea and green tea (GT3). Notably, the levels of quercetin (227.43 µg/g) and rutin (2617.13 µg/g) in RT were significantly higher than those of the other teas. As seen in Table 2, RT was composed of 10–30 times as much of quercetin and 3–5 times as much of rutin as the jasmine and green teas. Significantly higher amounts of apigenin and luteolin were found in AT while quercetin and quercetin glycosides in this tea sample followed the opposite trend. Notably, genistein is the flavonoid that was found only in AT and RT, but not detected in any of the teas. These are known as herbal teas, implying they did not stem from *Camellia sinensis.* AT, a popular drink in Vietnamese culture, is known as *trà atiso*, which is prepared from leaves, stalk and root of artichoke *(Cynara scolymus)*. According to the information about ingredients printed on the package, RT is a refreshing tea made from 85% sicklepod *(Senna obtusifolia)*, 12% Chinese liquorice *(Glycyrrhiza uralensis)* and 0.3% flowers of pagoda tree *(Sophora japonica)*. Reportedly, these plants did not contain catechins [32,33]. Perhaps because of this, the poor presence or absence of catechins in the studied herbal teas may be quite understandable. However, *Sophora japonica* is a highly rich source of quercetin and rutin [33], which is also confirmed here by the substantially high abundance of some flavonoids, such as quercetin and rutin, in RT.

### 3.3. Phenolic Acids

In general, significant differences in the concentrations of phenolic acids in the tea samples were noted (Table 3). As stated in the previous section, chlorogenic and gallic acids were the two predominating acids quantified in the present study. The levels of chlorogenic acid in the oolong tea (30.37 µg/g) and black tea (7.08 µg/g) were considerably lower than those in the jasmine teas (56.89–307.30 µg/g) and green teas (219.49–250.41 µg/g), with the exception of GT3. On the contrary, gallic acid exhibited an opposite trend: the jasmine, green and oolong teas contained lower amounts of gallic acid compared to the black tea (288.11 µg/g). The results are in agreement with what was reported by Jeszka-Skowron et al. (2018) on comparisons between Chinese green tea and Sri Lankan/Iranian black tea. In addition, the results revealed that the difference in gallic acid levels between the black and green teas was about 30%, approximate to those reported by Bae et al. (2015) [34]. However, Jeszka-Skowron et al. (2018) reported the level of gallic acid in black teas was two times higher than in green teas. Among the phenolic acids quantified, ferulic, syringic and vanillic acids were only present in a few of the samples. Furthermore, these compounds were detected at very low concentrations. It is noted that chlorogenic acid with the concentration of 341.88 µg/g was shown to abound in AT. Prior research showed that artichoke was rich in chlorogenic acid [32]. This could explain the higher level of this compound in AT compared to the other teas (Table 3). Along with chlorogenic acid, caffeic, ferulic, syringic and vanillic acids were found in AT at significantly higher levels compared to the teas.

### 3.4. Comparison between Solvent Extraction Methods

**Figure 2:**
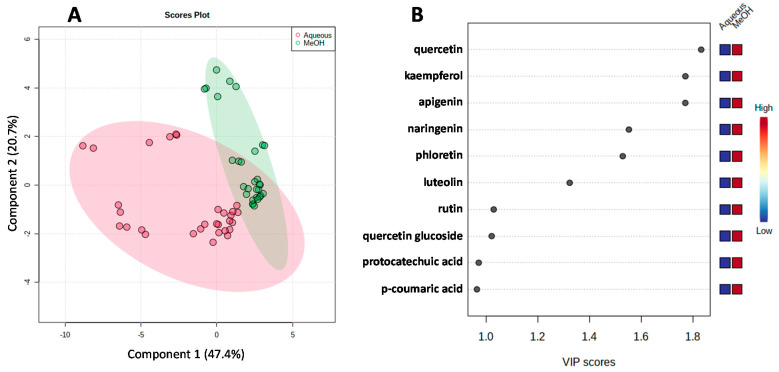
PLS-DA results using the phenolic data of the methanolic and aqueous extracts. Scores plot (**A**) and VIP score plot (**B**).

In the present study, along with methanol, we also used boiling water for extracting tea phenolics. This was to simulate how tea infusions were prepared from dry teas. The results of phenolic profile in the aqueous extracts compared to the methanolic extracts were displayed in Figure S1. The PLS-DA was used as a chemometric tool for identification of marker phenolics presenting differences in the two groups (i.e., methanolic and aqueous extracts). The scores plot (Figure 2A) shows a good group separation with 68.1% of the explained variability for the Component 1/Component 2 plot. The variable importance in projection (VIP) scores are the estimate of the importance of each variable in the PLS model. Among the important phenolics that contributed to the separation are quercetin, kaempferol, apigenin, naringenin, phloretin, luteolin, rutin, quercetin glucoside, protocatechuic acid and *p*-coumaric acid (Figure 2B). These compounds presented increased levels in the methanolic extracts. Recent research has revealed that methanolic extracts of tea are composed of higher phenolic contents compared to tea infusions [35,36]. Furthermore, methanol can enhance the extraction of catechin derivatives and flavonoid glycosides in green tea by at least 40% [36]. Despite more efficiency of methanol in extracting phenolics in tea, the methanolic extracts have not exhibited superior free radical scavenging potential to aqueous extracts. This corroborates that tea infusions not only provide a good source of bioactive phenolics but also exert high antioxidant activities of importance to human health.

### 3.5. Carotenoid Composition of the Teas

The results indicated that the teas differed significantly on the concentrations of four carotenoids (antheraxanthin, lutein, violaxanthin and zeaxanthin), chlorophyll A and chlorophyll B (Table 4). Lutein (3.50–103.68 µg/g) was found to be the most abundant carotenoids in all the teas. The results also showed that most of the examined jasmine and green teas contained significantly higher levels of lutein and violaxanthin compared to the oolong and black teas. Previously, green tea samples were reported to contain about 10 µg of lutein/g [37]. It has been shown that carotenoid oxidative degradation occurs during tea fermentation [38]. Because of this, it is understandable that oolong tea and black tea often comprise considerably lower amounts of carotenoids than green tea. Besides, there is much evidence to indicate that zeaxanthin is less stable against the oxidation than lutein [8]. This could in part contribute to the low zeaxanthin amounts measured for the oolong and black teas as shown in Table 4. Antheraxanthin was detected only in some of the jasmine and green teas but also at very low levels (0.06–0.1 µg/g). Chlorophylls A and B were found in most of the *Camellia sinensis* samples, except for GT3. Only chlorophyll B was detected in AT whereas the other examined carotenoids were not present in the two herbal teas. Previously, 38 carotenoids in green tea leaves were documented, proving this group of phytochemicals is abundant in tea [39].

In general, carotenoids are present in the teas at the levels comparable to those in commonly consumed oil nuts (i.e., pistachios, hazelnut, cashew and walnut) [40]. These constituents are well recognized micronutrients with bioactivities potentially important to human health, such as antioxidant activity, cardioprotective effect and protection against certain cancers [41]. A combined intake of these hydrophobic constituents and phenolics of tea could contribute more efficiently to prevention of lifestyle-related diseases.

### 3.6. Saccharides in the Teas

As seen in Table 4, sucrose appeared to be the most abundant saccharide in the teas, except for the black tea and refreshing tea. The levels of sucrose ranged between 5.17 and 25.95 mg/g. Glucose and fructose were found in all the samples, except for GT1, JT2 and RT. It was shown that in all samples where glucose was not detected, neither was fructose. Interestingly, RT did not comprise any of the examined saccharides although it tasted sweet. This was likely due to glycyrrhizin, a main sweet-tasting constituent, in *Glycyrrhiza uralensis* used as an ingredient of RT as stated earlier. The results in the present study also showed that these saccharides were present at the concentrations similar to those of green tea leaves reported by Shanmugavelan et al. (2013) [42]. The results of these tea constituents would give a better understanding of the beneficial effects of tea consumption on human health.

### 3.7. Compositional Variations of the Teas

**Figure 3:**
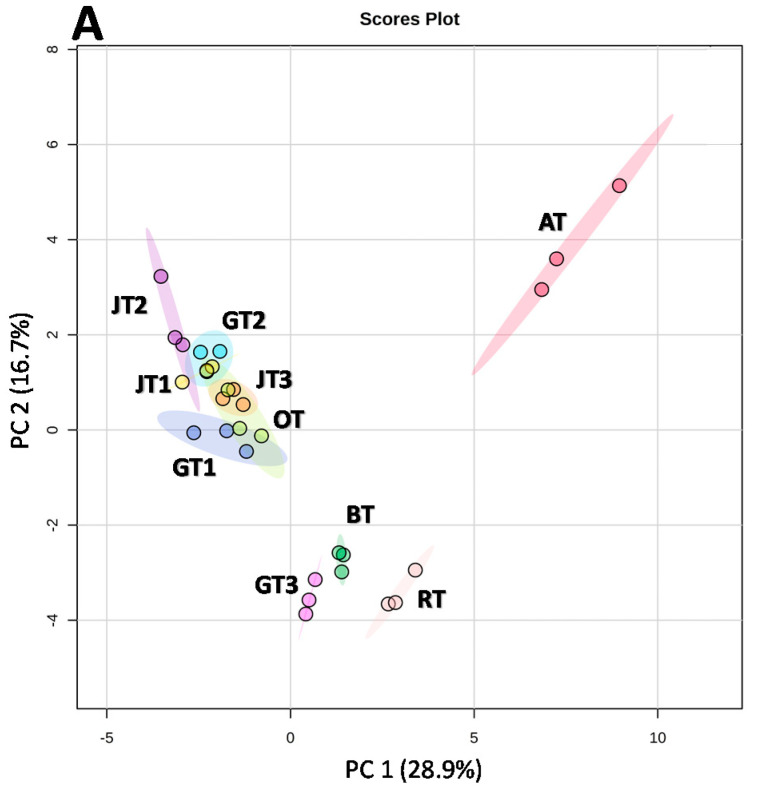

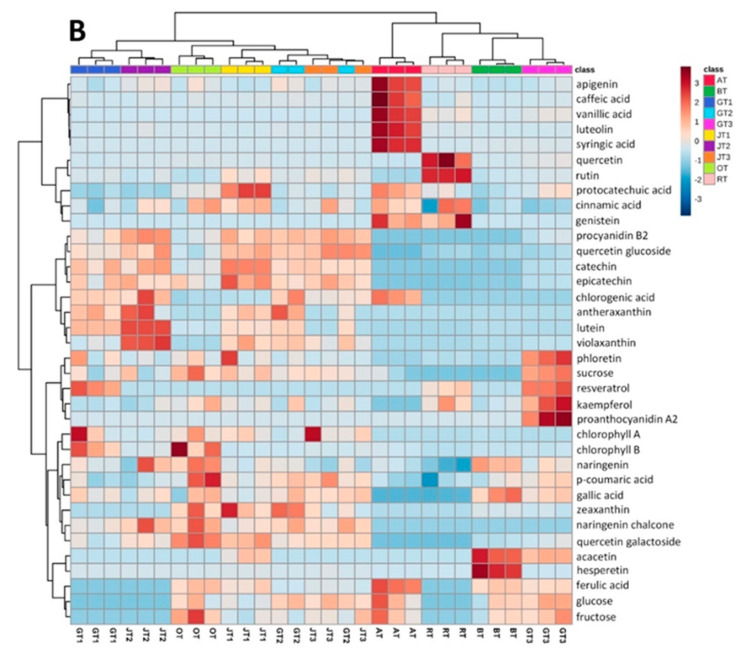
Principal component analysis (**A**) and heatmap (**B**) of phenolic, carotenoid and monosaccharide data of the studied teas and herbal teas.

Principal component analysis was employed to provide an overview of sample diversity. The PCA scores plot (Figure 3A) as an output of the analysis displays regional relationships between the tea samples. As illustrated in the figure, principal component 1 (PC 1) explains 28.9% of the total variability and is mainly contributed by apigenin, genistein, luteolin, catechins, procyanidin B2, quercetin glucoside, quercetin galactoside, caffeic, syringic and vanillic acids. As discussed above, these compounds were found in AT with concentrations significantly different from the other teas, particularly the jasmine, green and oolong teas. This unravels the distant positioning of AT from the other samples in the PCA scores plot. Principal component 2 explaining 16.7% is characterized by chlorogenic acid and kaempferol. Figure 3A also highlights GT3, BT and RT clustering separately from the others, and this can be explained by their considerably lower phenolic contents as described above. Conversely, GT1, GT2, OT and the jasmine teas with substantially higher phenolic contents are located on the upper-left region of the plot (quadrant II). The PCA scores plot shows 45.6% of the total variability in the dataset. Besides the PCA scores plot, a blue-red color-coded heatmap was used to visualize intuitively the concentrations of all the compounds determined in the study (Figure 3B). The red in the figure represents a higher content while the blue displays the opposite.

## 4. Conclusions

This is the first report to show the presence of phenolics, carotenoids, chlorophylls and saccharides in various Vietnamese teas. These constituents were successfully assessed using LC-MS/MS, HPLC-DAD and HPLC-ELSD analytical methods. In total, 27 phenolics, four carotenoids, two chlorophylls and three saccharides in ten *Camellia sinensis* teas and herbal teas have been quantitatively determined. These teas differed significantly on the levels of the examined compounds. Catechins and quercetin glycosides were identified as the most abundant flavonoids in the teas. Chlorogenic and gallic acids were found at the highest levels among the phenolic acids. In most cases, the levels of these compounds, except for gallic acid, followed the order: jasmine/green teas > oolong tea > black tea. Significantly, differences in phenolic contents between the *Camellia sinensis* teas and herbal teas were observed. The highest concentrations of chlorogenic, caffeic, ferulic, syringic and vanillic acids were determined in AT while that of rutin was found in RT. Genistein was present only in the herbal teas. Lutein and sucrose were found to be the most abundant carotenoid and saccharide in the teas, respectively.

The use of PLS-DA as a mathematical tool allowed differentiation of tea infusions from methanolic extracts in respect to phenolic profile. PCA graphically presented the differences between the teas and herbal teas. This plot also illustrated similarities and dissimilarities among the jasmine, green, oolong and black teas.

The study shows that jasmine/green and oolong teas originating in Vietnam are rich sources of phenolic acids, flavonoids and carotenoids. These constituents have aroused considerable attention due to their bioactivities of potential importance to human health. The study also indicates herbal teas are medicinally important products and this could help promote the consumption of herbal teas as well as improve their reputation in the world market. Future research should be focused on the aroma and taste as well as the phytochemical profile of Vietnamese teas during processing.

## Figures and Tables

**Table 1:** Summary of the teas examined in this study.

	Abbreviations	Vietnamese Name	Packaging	Purchase Location	Origin
Jasmine tea 1	JT1	*trà lài*	loose tea	Vietnam	Lam Dong, Vietnam
Jasmine tea 2	JT2		loose tea	Vietnam	Thai Nguyen, Vietnam
Jasmine tea 3	JT3		loose tea	USA	China
Green tea 1	GT1	*trà xanh*	loose tea	Vietnam	Thai Nguyen, Vietnam
Green tea 2	GT2		loose tea	Vietnam	Thai Nguyen, Vietnam
Green tea 3	GT3		teabag	USA	China
Oolong tea	OT	*trà ô long*	loose tea	Vietnam	Lam Dong, Vietnam
Black tea	BT	*trà đen*	teabag	USA	Sri Lanka
Artichoke tea	AT	*trà atisô*	teabag	Vietnam	Lam Dong, Vietnam
Refreshing tea	RT	*trà thanh nhiệt*	teabag	Vietnam	Lam Dong, Vietnam

**Table 2:** Flavonoids (µg/g) in the studied teas.

	Acacetin *	Apigenin *	Catechin	Epicatechin	Genistein *	Hesperetin *	Kaempferol	Luteolin	Naringenin *
JT1	3.32 ± 0.83 b	56.12 ± 1.59 b	636.58 ± 4.95 a	546.77 ± 57.33 a	nd	9.46 ± 1.39 b	7.77 ± 0.56 bc	0.70 ± 0.16 b	75.68 ± 5.77 abc
JT2	nd	90.93 ± 5.55 b	462.73 ± 85.26 ab	345.23 ± 53.66 ab	nd	5.85 ± 0.44 b	4.92 ± 1.51 bc	3.06 ± 1.22 b	104.07 ± 31.96 ab
JT3	nd	71.66 ± 9.61 b	364.68 ± 22.09 bc	369.06 ± 56.92 ab	nd	9.16 ± 2.28 b	5.66 ± 0.51 bc	1.88 ± 0.38 b	54.84 ± 2.64 bc
GT1	nd	56.01 ± 8.74 b	419.17 ± 62.65 b	316.11 ± 68.23 bc	nd	5.69 ± 0.22 b	4.73 ± 0.36 bc	1.02 ± 0.08 b	78.56 ± 5.13 abc
GT2	nd	90.60 ± 8.53 b	358.66 ± 24.77 bc	319.09 ± 26.93 bc	nd	5.47 ± 0.03 b	8.39 ± 2.54 bc	2.33 ± 0.27 b	81.94 ± 1.44 abc
GT3	4.83 ± 0.29 b	65.70 ± 6.06 b	122.44 ± 6.29 d	115.78 ± 3.23 c	nd	nd	20.96 ± 3.47 a	0.99 ± 0.16 b	83.69 ± 7.20 abc
OT	nd	80.18 ± 14.07 b	201.88 ± 21.23 cd	258.03 ± 55.89 bc	nd	5.98 ± 0.21 b	10.86 ± 2.31 b	1.48 ± 0.28 b	126.21 ± 13.69 a
BT	8.80 ± 0.76 a	51.57 ± 0.41 b	7.97 ± 0.08 e	25.24 ± 0.64 d	nd	174.67 ± 13.54 a	5.39 ± 0.83 bc	0.63 ± 0.08 b	117.94 ± 4.63 a
AT	nd	290.13 ± 27.06 a	nd	0.77 ± 0.68 e	52.23 ± 11.09	nd	0.74 ± 0.11 c	75.47 ± 6.09 a	67.74 ± 3.59 abc
RT	nd	64.47 ± 3.58 b	0.20 ± 0.15 e	1.12 ± 0.76 e	53.06 ± 20.35	nd	12.21 ± 2.57 ab	2.53 ± 0.49 b	30.17 ± 6.36 c
	**Naringenin** chalcone *	**Procyanidin** **A2**	**Procyanidin** **B2**	Phloretin *	**Quercetin** **glucoside**	**Quercetin** **galactoside**	**Quercetin**	Resveratrol *	**Rutin**
JT1	5.91 ± 0.84 bc	nd	360.90 ± 41.85 a	2.46 ± 1.30 ab	515.02 ± 28.43 ab	180.70 ± 15.63 b	12.55 ± 1.25 b	nd	760.01 ± 92.83 b
JT2	12.48 ± 2.91 a	nd	466.31 ± 18.56 a	0.85 ± 0.20 b	501.26 ± 66.93 ab	115.92 ± 7.74 cd	11.17 ± 0.85 b	nd	334.92 ± 61.73 cde
JT3	7.47 ± 0.61 ab	nd	377.65 ± 36.56 a	0.88 ± 0.08 b	585.21 ± 54.48 a	136.07 ± 2.05 bc	7.53 ± 0.66 b	nd	523.51 ± 47.78 bc
GT1	5.45 ± 0.61 bc	nd	241.10 ± 29.20 b	1.96 ± 0.82 ab	338.28 ± 17.43 bc	75.60 ± 13.68 ed	6.69 ± 1.80 b	4.06 ± 0.51 a	408.94 ± 15.27 cde
GT2	11.14 ± 1.72 ab	nd	367.69 ± 17.36 a	0.94 ± 0.20 b	519.83 ± 68.69 ab	119.96 ± 10.45 cd	9.24 ± 0.99 b	nd	506.41 ± 58.00 cd
GT3	nd	1.11 ± 0.21	144.27 ± 17.82 b	4.83 ± 0.46 a	136.99 ± 15.86 de	80.47 ± 7.40 ed	22.37 ± 2.44 b	4.52 ± 0.28 a	388.16 ± 22.97 cde
OT	12.71 ± 3.35 a	nd	164.11 ± 20.17 b	1.53 ± 0.40 b	201.93 ± 32.46 cd	241.08 ± 15.95 a	14.95 ± 0.24 b	nd	195.93 ± 26.08 e
BT	nd	nd	7.99 ± 1.68 c	0.34 ± 0.04 b	107.27 ± 17.17 de	53.70 ± 2.53 e	7.97 ± 0.37 b	nd	269.24 ± 43.08 de
AT	nd	nd	nd	nd	0.74 ± 0.04 f	1.99 ± 0.39 f	1.00 ± 0.12 c	nd	18.37 ± 2.47 f
RT	nd	nd	0.34 ± 0.23 c	0.48 ± 0.03 b	113.84 ± 10.82 de	0.05 ± 0.02 f	227.43 ± 35.02 a	1.87 ± 0.28 b	2617.13 ± 41.85 a

*: µg/100 g; nd: not detected. Different letters for the same flavonoids show statistically significant differences among the extracts (*p* < 0.05).

**Table 3:** Phenolic acids (µg/g) in the studied teas.

	Caffeic Acid	Chlorogenic Acid	Cinnamic Acid *	*p*-Coumaric Acid	Ferulic Acid *	Gallic Acid	Protocatechuic Acid	Syringic Acid	Vanillic Acid *
JT1	1.51 ± 0.11 b	56.89 ± 1.98 cd	52.69 ± 3.56	4.17 ± 0.26 abc	60.56 ± 7.11 b	138.58 ± 9.42 b	63.82 ± 4.46 a	nd	nd
JT2	1.28 ± 0.30 b	307.30 ± 86.99 ab	13.22 ± 1.81	3.02 ± 0.41 bc	nd	150.26 ± 28.41 ab	7.69 ± 0.83 de	nd	nd
JT3	1.57 ± 0.06 b	147.76 ± 10.17 c	43.51 ± 9.06	5.95 ± 0.69 ab	36.23 ± 2.22 c	199.93 ± 14.78 a	15.29 ± 1.62 cde	nd	nd
GT1	1.14 ± 0.22 b	219.49 ± 4.90 bc	30.98 ± 5.60	3.16 ± 0.63 bc	nd **	176.23 ± 22.34 a	8.00 ± 2.13 de	nd	nd
GT2	1.79 ± 0.17 b	250.41 ± 63.17 ab	35.06 ± 2.60	5.20 ± 0.40 abc	32.48 ± 2.00 c	210.91 ± 25.50 a	17.39 ± 1.97 cde	nd	nd
GT3	1.19 ± 0.15 b	8.63 ± 0.59 e	24.86 ± 1.50	4.96 ± 0.40 abc	62.95 ± 3.52 b	201.03 ± 17.78 a	24.75 ± 3.55 c	0.31 ± 0.01 b	1.25 ± 0.13 b
OT	1.30 ± 0.38 b	30.37 ± 8.16 d	49.70 ± 10.19	7.35 ± 1.85 a	76.71 ± 7.68 b	184.70 ± 66.41 a	21.97 ± 0.93 cd	nd	nd
BT	0.69 ± 0.13 b	7.08 ± 1.64 e	27.85 ± 3.62	3.82 ± 0.49 abc	73.28 ± 4.96 b	288.11 ± 44.43 a	11.66 ± 2.32 cde	0.28 ± 0.02 b	0.19 ± 0.02 b
AT	20.12 ± 3.11 a	341.88 ± 34.32 a	52.01 ± 4.28	2.66 ± 0.34 bc	130.49 ± 7.65 a	1.33 ± 0.24 c	47.06 ± 4.62 b	12.11 ± 0.84 a	12.71 ± 1.23 a
RT	1.17 ± 0.98 b	1.44 ± 0.52 e	50.31 ± 20.08	1.96 ± 0.97 c	nd	4.03 ± 1.94 c	20.44 ± 3.07 cde	nd	1.70 ± 0.32 b

*: µg/100 g; nd: not detected. Different letters for the same phenolic acids show statistically significant differences among the extracts (*p* < 0.05).

**Table 4:** Carotenoid (µg/g) and saccharide contents (mg/g) in the teas and herbal teas.

	Antheraxanthin	Lutein	Violaxanthin	Zeaxanthin	Chlorophyll A	Chlorophyll B	Sucrose	Glucose	Fructose
JT1	0.06 ± 0.00	35.33 ± 1.29 d	1.24 ± 0.23 b	0.55 ± 0.20 a	0.51 ± 0.11	0.26 ± 0.03 b	17.25 ± 2.45 abc	2.05 ± 0.26	1.35 ± 0.34 bc
JT2	0.1 ± 0.04	103.68 ± 0.14 a	2.53 ± 0.08 a	nd	0.19 ± 0.05	0.47 ± 0.06 b	10.74 ± 4.42 bcd	nd	nd
JT3	nd	4.32 ± 0.21 f	0.62 ± 0.10 c	0.15 ± 0.08 ab	1.01 ± 0.51	0.45 ± 0.06 b	13.17 ± 1.63 abcd	3.63 ± 0.49	2.60 ± 0.54
GT1	0.07 ± 0.00	56.53 ± 0.69 b	0.39 ± 0.08 cd	nd	0.96 ± 0.54	2.79 ± 0.72 a	9.23 ± 3.25 bcd	nd	nd
GT2	0.09 ± 0.03	43.75 ± 1.46 c	1.23 ± 0.04 b	0.54 ± 0.15 a	0.21 ± 0.07	0.28 ± 0.02 b	13.04 ± 0.88 abcd	4.01 ± 0.21	1.50 ± 0.28 bc
GT3	nd	3.50 ± 0.06 f	0.19 ± 0.00 cd	0.08 ± 0.02 b	nd	0.18 ± 0.02 b	25.95 ± 1.67 a	4.53 ± 0.52	3.63 ± 0.68
OT	nd	11.87 ± 0.74 e	nd	0.46 ± 0.15 a	0.65 ± 0.24	3.70 ± 1.19 a	22.04 ± 4.43 ab	3.24 ± 0.85	4.69 ± 1.04 a
BT	nd	5.40 ± 0.03 f	0.08 ± 0.00 d	0.12 ± 0.00 ab	0.04 ± 0.00	0.16 ± 0.00 b	nd	2.94 ± 0.84	2.11 ± 0.62
AT	nd	nd	nd	nd	nd	0.31 ± 0.07 b	5.17 ± 2.75 cd	4.70 ± 1.33	3.71 ± 1.14
RT	nd	nd	nd	nd	nd	nd	nd	nd	nd

nd: not detected. Different letters for the same carotenoids or saccharides show statistically significant differences among the extracts (*p* < 0.05).

## Data Availability

The data generated or analyzed during the study are included in the article and its Supplementary material file.

## Supplementary Materials

The following are available online, Table S1. MRM transitions of the phenolics screened in the study, Table S2. Retention times, calibration equations, correlation coefficients, sensitivity and repeatability of the monitored phenolics, Figure S1. Comparisons of phenolic profiles between the methanol and aqueous extracts.

## References

[1] Hayat K., Iqbal H., Malik U., Bilal U., Mushtaq S. (2015). Tea and its consumption: Benefits and risks. Crit. Rev. Food Sci. Nutr..

[2] Agati G., Azzarello E., Pollastri S., Tattini M. (2012). Flavonoids as antioxidants in plants: Location and functional significance. Plant. Sci..

[3] Li W., He N., Tian L., Shi X., Yang X. (2016). Inhibitory effects of polyphenol-enriched extract from Ziyang tea against human breast cancer MCF-7 cells through reactive oxygen species-dependent mitochondria molecular mechanism. J. Food Drug Anal..

[4] Yang C.S., Wang H. (2016). Cancer preventive activities of tea catechins. Molecules.

[5] Yang C.S., Wang H., Sheridan Z.P. (2018). Studies on prevention of obesity, metabolic syndrome, diabetes, cardiovascular diseases and cancer by tea. J. Food Drug Anal..

[6] Liu Z., Zhou T., Ziegler A.C., Dimitrion P., Zuo L. (2017). Oxidative stress in neurodegenerative diseases: From molecular mechanisms to clinical applications. Oxid. Med. Cell. Longev..

[7] Chacko S.M., Thambi P.T., Kuttan R., Nishigaki I. (2010). Beneficial effects of green tea: A literature review. Chinese Med..

[8] Ho C.-T., Zheng X., Li S. (2015). Tea aroma formation. Food Sci. Hum. Wellness.

[9] Stahl W., Sies H. (2005). Bioactivity and protective effects of natural carotenoids. Biochim. Biophys. Acta Mol. Basis Dis..

[10] Wang Y., Yang Z., Wei X. (2010). Sugar compositions, α-glucosidase inhibitory and amylase inhibitory activities of polysaccharides from leaves and flowers of Camellia sinensis obtained by different extraction methods. Int. J. Biol. Macromol..

[11] Hidalgo-Cantabrana C., Delgado S., Ruiz L., Ruas-Madiedo P., Sánchez B., Margolles A. (2017). Bifidobacteria and their health-promoting effects. Microbiol. Spectr..

[12] Khoi N.V., Lan C.H., Huong T.L. (2015). Vietnam tea industry-an analysis from value chain approach. Int. J. Manag. Value Supply Chain..

[13] Tuan N.-A. (2020). Vietnamese Tea Exporting and Forecasting to 2030. Vietnam. J. Agric. Sci..

[14] Vuong Q.V., Nguyen V., Golding J.B., Roach P.D. (2011). The content of bioactive constituents as a quality index for Vietnamese teas. Int. Food Res. J..

[15] Nguyen C.T., Lee A.H., Pham N.M., Do V.V., Ngu N.D., Tran B.Q., Binns C. (2018). Habitual tea drinking associated with a lower risk of type 2 diabetes in Vietnamese adults. Asia Pac. J. Clin. Nutr..

[16] Van Dong Hoang A.H.L., Pham N.M., Xu D., Binns C.W. (2016). Habitual tea consumption reduces prostate cancer risk in Vietnamese Men: A case-control study. Asian Pac. J. Cancer Prev..

[17] Dautermann O., Lohr M. (2017). A functional zeaxanthin epoxidase from red algae shedding light on the evolution of light-harvesting carotenoids and the xanthophyll cycle in photosynthetic eukaryotes. Plant J..

[18] Chong J., Soufan O., Li C., Caraus I., Li S., Bourque G., Wishart D.S., Xia J. (2018). MetaboAnalyst 4.0: Towards more transparent and integrative metabolomics analysis. Nucleic Acids Res..

[19] Yang J., Liu R.H. (2013). The phenolic profiles and antioxidant activity in different types of tea. Int. J. Food Sci. Technol..

[20] Muhammad H.F.L., Dickinson K.M. (2019). Nutrients, Energy Values and Health Impact of Conventional Beverages.

[21] Jeszka-Skowron M., Zgoła-Grześkowiak A., Frankowski R. (2018). Cistus incanus a promising herbal tea rich in bioactive compounds: LC–MS/MS Determination of catechins, flavonols, phenolic acids and alkaloids—A comparison with Camellia sinensis, Rooibos and Hoan ngoc herbal tea. J. Food Compos. Anal..

[22] Jiang H., Yu F., Qin L., Zhang N., Cao Q., Schwab W., Li D., Song C. (2019). Dynamic change in amino acids, catechins, alkaloids, and gallic acid in six types of tea processed from the same batch of fresh tea (Camellia sinensis L.) leaves. J. Food Compos. Anal..

[23] Pinto G., Illiano A., Carpentieri A., Spinelli M., Melchiorre C., Fontanarosa C., Serio M.d., Amoresano A. (2020). Quantification of polyphenols and metals in Chinese tea infusions by mass spectrometry. Foods.

[24] Dai W., Xie D., Lu M., Li P., Lv H., Yang C., Peng Q., Zhu Y., Guo L., Zhang Y. (2017). Characterization of white tea metabolome: Comparison against green and black tea by a nontargeted metabolomics approach. Food Res. Int..

[25] Wu C., Xu H., Héritier J., Andlauer W. (2012). Determination of catechins and flavonol glycosides in Chinese tea varieties. Food Chem..

[26] Jiang H., Engelhardt U.H., Thräne C., Maiwald B., Stark J. (2015). Determination of flavonol glycosides in green tea, oolong tea and black tea by UHPLC compared to HPLC. Food Chem..

[27] Tan J., Dai W., Lu M., Lv H., Guo L., Zhang Y., Zhu Y., Peng Q., Lin Z. (2016). Study of the dynamic changes in the non-volatile chemical constituents of black tea during fermentation processing by a non-targeted metabolomics approach. Food Res. Int..

[28] Zeng C., Lin H., Liu Z., Liu Z. (2020). Metabolomics analysis of Camellia sinensis with respect to harvesting time. Food Res. Int..

[29] Ramakrishnan P., Rangiah K. (2016). A UHPLC-MS/SRM method for analysis of phenolics from Camellia sinensis leaves from Nilgiri hills. Anal. Methods.

[30] Konieczynski P., Viapiana A., Wesolowski M. (2017). Comparison of infusions from black and green teas (Camellia sinensis L. Kuntze) and erva-mate (Ilex paraguariensis A. St.-Hil.) based on the content of essential elements, secondary metabolites, and antioxidant activity. Food Anal. Methods.

[31] Su X., Wang W., Xia T., Gao L., Shen G., Pang Y. (2018). Characterization of a heat responsive UDP: Flavonoid glucosyltransferase gene in tea plant (Camellia sinensis). PLoS ONE.

[32] Sa R.R., Matos R.A., Silva V.C., da Cruz Caldas J., da Silva Sauthier M.C., dos Santos W.N.L., Magalhães H.I.F., Júnior A.d.F.S. (2017). Determination of bioactive phenolics in herbal medicines containing Cynara scolymus, Maytenus ilicifolia Mart ex Reiss and Ptychopetalum uncinatum by HPLC-DAD. Microchem. J..

[33] Chen G.-L., Chen S.-G., Xiao Y., Fu N.-L. (2018). Antioxidant capacities and total phenolic contents of 30 flowers. Ind. Crops Prod..

[34] Bae I.K., Ham H.M., Jeong M.H., Kim D.H., Kim H.J. (2015). Simultaneous determination of 15 phenolic compounds and caffeine in teas and mate using RP-HPLC/UV detection: Method development and optimization of extraction process. Food Chem..

[35] Anand J., Upadhyaya B., Rawat P., Rai N. (2015). Biochemical characterization and pharmacognostic evaluation of purified catechins in green tea (Camellia sinensis) cultivars of India. 3 Biotech.

[36] Barreira S., Moutinho C., Silva A.M.N., Neves J., Seo E.-J., Hegazy M.-E.F., Efferth T., Gomes L.R. (2021). Phytochemical characterization and biological activities of green tea (Camellia sinensis) produced in the Azores, Portugal. Phytomedicine Plus.

[37] Loranty A., Rembiałkowska E., Rosa E.A.S., Bennett R.N. (2010). Identification, quantification and availability of carotenoids and chlorophylls in fruit, herb and medicinal teas. J. Food Compos. Anal..

[38] Ravichandran R. (2002). Carotenoid composition, distribution and degradation to flavour volatiles during black tea manufacture and the effect of carotenoid supplementation on tea quality and aroma. Food Chem..

[39] Suzuki Y., Shioi Y. (2003). Identification of chlorophylls and carotenoids in major teas by high-performance liquid chromatography with photodiode array detection. J. Agric. Food Chem..

[40] Stuetz W., Schlörmann W., Glei M. (2017). B-vitamins, carotenoids and α-/γ-tocopherol in raw and roasted nuts. Food Chem..

[41] Tanaka T., Shnimizu M., Moriwaki H. (2012). Cancer chemoprevention by carotenoids. Molecules.

[42] Shanmugavelan P., Kim S.Y., Kim J.B., Kim H.W., Cho S.M., Kim S.N., Kim S.Y., Cho Y.S., Kim H.R. (2013). Evaluation of sugar content and composition in commonly consumed Korean vegetables, fruits, cereals, seed plants, and leaves by HPLC-ELSD. Carbohydr. Res..

