# Supplementary material for: Phenolic, Carotenoid and Saccharide Compositions of Vietnamese Camellia sinensis Teas and Herbal Teas
Source: Molecules. 2021 Oct 27;26(21):6496. doi: 10.3390/molecules26216496 (PMC8587765; doi:10.3390/molecules26216496)
Supplement: Supplementary file 1 [file molecules-26-06496-s001.zip › molecules-1428283-supplementary.pdf]

molecules

Supplementary Material

Phenolic, Carotenoid and Saccharide Compositions of Vietnamese *Camellia sinensis* Teas and Herbal Teas

Danh C. Vu [1,*] and Sophie Alvarez [2]

[1] Institute of Applied Technology, Thu Dau Mot University, Binh Duong Province, Vietnam
[2] Proteomics and Metabolomics Core Facility, Center for Biotechnology, University of Nebraska, Lincoln, Nebraska 68503, USA; salvarez@unl.edu
* Correspondence: danhvc@tdmu.edu.vn; Tel.: +84-274-38-22-518

Table S1. MRM transitions of the phenolics screened in the study.

Compound	ESI	Molecular Ion	Product Ion	DP, volts	EP, volts	CE, eV	CXP, volts
Acacetin	−	283	268	−87	−9	−33	−36
Apigenin	−	269	117	−90	−11	−46	−13
Caffeic acid	−	179	135	−62	−7	−19	−8
Catechin	−	289	245	−97	−11.3	−22.1	−14.7
Chlorogenic acid	−	353	191	−76.5	−3.9	−20	−21.3
Cinnamic acid	−	147	103	−65	−10	−12	−12
Cyanidin chloride	+	287	287	95	5	41	12
Daidzein	−	253	224	−106.8	−12.2	−36.4	−12
Delphinidin chloride	+	303	229	101.3	3.9	43	14
Epicatechin	−	289	125	−74	−10.2	−29.7	−21.3
Ferulic acid	−	193	134	−62	−7	−23	−8
Gallic acid	−	169	125	−61	−9	−20	−7
Genistein	−	269	133	−86	−12	−39	−7
Hesperetin	+	303	177	91	6	24	11
Kaempferol	−	285	117	−133	−5	−52	−14
Luteolin	−	285	133	−97	−11	−48	−8
Naringenin	−	271	151	−99	−13	−24	−12
Naringenin chalcone	−	271	151	−83	−8	−23	−12
p-Coumaric acid	−	163	119	−42	−9	−20	−15
Phloretin	−	273	167	−75	−3	−22	−14
Proanthocyanidin A2	−	575	285	−112.7	−6	−34.5	−35.1
Procyanidin B2	−	577	289	−87.6	−3.9	−30.9	−18.2
Protocatechuic acid	−	153	109	−73	−12.8	−20.7	−12.3
Quercetin	−	301	151	−95	−11	−29	−11
Quercetin-3-galactoside	−	463	300	−78.3	−10	−40.5	−8.9
Quercetin-3-glucoside	−	463	300	−90	−12	−29	−12.9
Resveratrol	−	227	143	−77	−7	−37	−17
Rutin	−	609	300	−138	−9.8	−47	−32.9
Syringic acid	−	197	121	−87	−12	−21	−14
Vanillic acid	−	167	123	−8.6	−4.1	−16.7	−14.1

Table S2. Retention times, calibration equations, correlation coefficients, sensitivity and repeatability of the monitored phenolics.

Compound	Retention Time, min	Linear Equation	Correlation Coefficient R^2	LOD, µg/L	LOQ, µg/L	Repeatability, %	
						Intraday	Interday
Gallic acid	1.69	y = 4,481,700x	0.9996	0.49	1.63	1.7	10.4
Protocatechuic acid	2.89	y = 2,010,835x	0.9999	0.12	0.40	2.1	4.3
Catechin	4.12	y = 1,009,730x	0.9994	0.33	1.09	0.5	3.5
Chlorogenic acid	4.20	y = 4,996,159x	0.9991	0.12	0.36	0.5	4.4
Procyanidin B2	4.64	y = 2,350,135x	0.9996	0.17	0.56	0.8	1.3
Vanillic acid	5.01	y = 449,059x	0.9996	0.75	2.47	2.0	4.5
Caffeic acid	5.11	y = 6,055,373x	0.9996	0.14	0.45	1.0	4.1
Syringic acid	5.32	y = 271,734x	0.9982	0.33	1.08	4.4	7.1
Epicatechin	5.41	y = 367,725x	0.9995	0.57	1.89	1.0	1.0
p-Coumaric acid	6.92	y = 5,646,083x	0.9999	0.23	0.75	1.0	4.0
Ferulic acid	7.73	y = 1,995,261x	0.9991	0.19	0.63	1.4	4.9
Rutin	7.73	y = 4,882,345x	0.9999	0.19	0.64	1.7	2.4
Quercetin galactoside	8.00	y = 8,923,578x	0.9991	0.29	0.97	0.4	5.1
Procyanidin A2	8.08	y = 1,321,803x	0.9984	0.17	0.57	1.0	1.1
Quercetin glucoside	8.23	y = 4,360,387x	0.9999	0.15	0.51	1.1	2.2
Resveratrol	10.84	y = 2,229,418x	0.9998	0.05	0.17	1.2	5.3
Daidzein	11.12	y = 1,709,516x	0.9965	0.02	0.07	1.4	6.1
Luteolin	11.43	y = 6,622,982x	0.9989	0.04	0.13	1.6	8.3
Quercetin	11.47	y = 8,768,515x	0.9979	0.03	0.10	0.8	7.2
Cinnamic acid	11.73	y = 1,240,614x	0.9995	0.31	1.03	2.1	7.1
Naringenin chalcone	11.95	y = 9,245,948x	0.9993	0.07	0.24	4.3	8.3
Phloretin	12.00	y = 25,489,951x	0.9999	0.01	0.03	1.5	9.3
Naringenin	12.05	y = 6,140,859x	0.9984	0.05	0.16	3.5	6.2
Apigenin	12.09	y = 5,146,699x	0.9994	0.03	0.11	0.4	5.9
Genistein	12.10	y = 1,299,352x	0.9962	0.07	0.22	0.3	5.6
Kaempferol	12.22	y = 318,331x	0.9996	0.17	0.55	1.6	5.4
Hesperetin	12.28	y = 4,828,599x	0.9981	0.01	0.03	2.8	12.2
Acacetin	13.60	y = 36,608,428x	0.9999	0.04	0.12	1.6	5.6

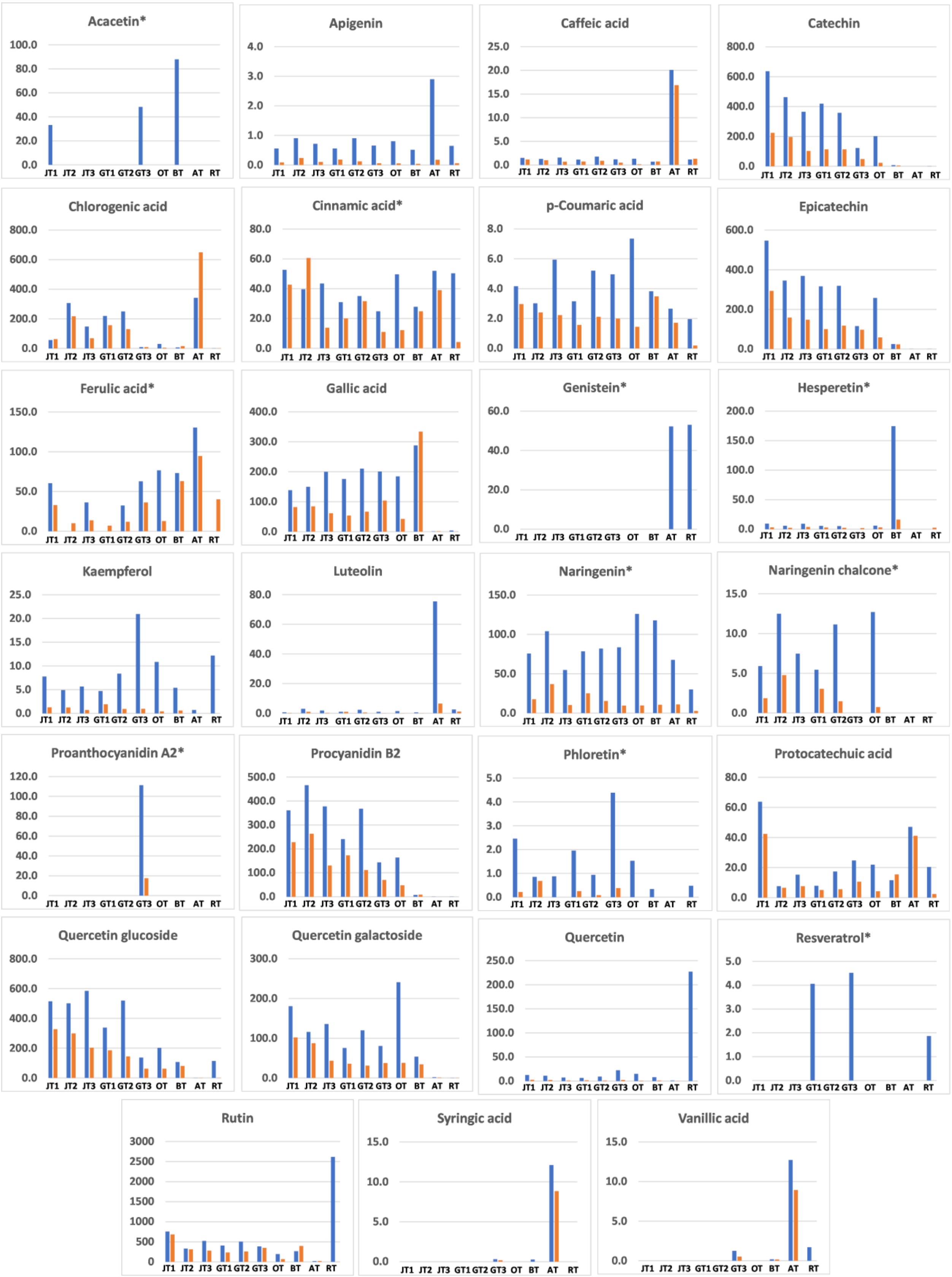

Figure S1. Comparisons of phenolic profiles between the methanol and aqueous extracts. Blue and red bars represent methanolic and water extracts, respectively. Y-axis indicates the concentration of phenolics (µg/g DW). * indicates the compound concentration reported in µg/100 g of dry weight. Abbreviations: JT1—jasmine tea 1; JT2—jasmine tea 2; JT3—jasmine tea 3; GT1—green tea 1; GT2—green tea 2; GT3—green tea 3; OT—oolong tea; BT—black tea; AT—artichoke tea; RT—refreshing tea.
